# Not Only a Weed Plant—Biological Activities of Essential Oil and Hydrosol of *Dittrichia viscosa* (L.) Greuter

**DOI:** 10.3390/plants10091837

**Published:** 2021-09-04

**Authors:** Elma Vuko, Valerija Dunkić, Ana Maravić, Mirko Ruščić, Marija Nazlić, Mila Radan, Ivica Ljubenkov, Barbara Soldo, Željana Fredotović

**Affiliations:** 1 Department of Biology, Faculty of Science, University of Split, R. Boškovića 33, 21000 Split, Croatia; elma@pmfst.hr (E.V.); dunkic@pmfst.hr (V.D.); amaravic@pmfst.hr (A.M.); mrus@pmfst.hr (M.R.); mnazlic@pmfst.hr (M.N.); 2 Department of Biochemistry, Faculty of Chemistry and Technology, University of Split, R. Boškovića 33, 21000 Split, Croatia; mradan@ktf-split.hr; 3 Department of Chemistry, Faculty of Science, University of Split, R. Boškovića 33, 21000 Split, Croatia; iljubenk@pmfst.hr (I.L.); barbara@pmfst.hr (B.S.)

**Keywords:** *Dittrichia viscosa*, essential oil, hydrosol, GSH, antimicrobial, antiproliferative, antiphytoviral activity

## Abstract

With the increasing interest in obtaining biologically active compounds from natural sources, *Dittrichia viscosa* (L.) Greuter (Asteraceae) came into our focus as a readily available and aromatic wild shrub widely distributed in the Mediterranean region. This work provides a phytochemical profile of *D. viscosa* in terms of parallel chemical composition in the lipophilic fraction (essential oil) and the water fraction (hydrosol). GC-MS analysis identified *1,8*-cineole, caryophyllene oxide, α-terpenyl acetate, and *α*-muurolol as the major components of the essential oil, while in the hydrosol *p*-menth-1-en-9-ol, *1,8*-cineole, linalool, *cis*-sabinene hydrate, and *α*-muurolol were the major volatile components. *3,4*-Dihydroxybenzoic acid was found to be the predominant compound in the hydrosol composition by HPLC analysis. The antimicrobial potential of both extracts was evaluated against thirteen opportunistic pathogens associated with common skin and wound infections and emerging food spoilage microorganisms. The antimicrobial activity of the essential oil suggests that the volatiles of *D. viscosa* could be used as novel antimicrobial agents. The antiproliferative results of *D. viscosa* volatiles are also new findings, which showed promising activity against three cancer cell lines: HeLa (cervical cancer cell line), HCT116 (human colon cancer cell line), and U2OS (human osteosarcoma cell line). The decrease in GSH level observed in hydrosol-treated HeLa cells suggests oxidative stress as a possible mechanism of the antiproliferative effect of hydrosol on tumor cells. The presented results are also the first report of significant antiphytoviral activity of hydrosol against tobacco mosaic virus (TMV) infection. Based on the results, *D. viscosa* might have the potential to be used in crop protection, as a natural disinfectant and natural anticancer agent.

## 1. Introduction

Plants are one of the most important sources of a variety of bioactive compounds that make them useful in daily life. Accordingly, a large number of plant species have recently become the focus of phytochemical and pharmacological studies. Considering that only 1–10% of plant species have been studied chemically and pharmacologically for their potential medicinal value [1], it is clear that plants are an under-researched natural source of bioactive compounds. Therefore, the study of their metabolites and biological effects will continue to be the focus of scientific interest with the aim of finding bioactive natural compounds and further development of alternative green and sustainable technologies that reduce or eliminate the use of hazardous substances in everyday life.

The Mediterranean climate favors the growth of a large number of plant species, many of which are aromatic plants used in folk medicine and nutrition. *Dittrichia viscosa* (L.) Greuter (syn. *Inula viscosa* L. (Aiton)) is a weed plant with numerous biological activities. It is a perennial herbaceous plant of the Asteraceae family. The plant is erect, 40–140 cm tall, and branched, with a prominent central shoot. The leaves are stalkless, alternate on the stem, and have a serrated margin directed towards the leaf tip. The yellow flower heads are 20–22 mm in size. The whole plant, especially the leaves, is covered with glandular hairs that secrete a sticky, aromatic-smelling resin [2,3,4,5]. Among the Mediterranean wild species, *D. viscosa* has been shown to be a remarkable source of potential bioactive metabolites that could find application in agriculture and other fields [6]. Folk medicine describes the use of this plant for the treatment of various diseases such as bronchitis and diabetes [1], as well as for its antipyretic, anti-inflammatory, and anthelmintic properties [7].

As an aromatic species with intense odor, this plant has been the subject of phytochemical profiling of volatile components performed by steam distillation, solvent extraction, and extraction by ultrasonic distillation [8,9,10]. The composition of the essential oil of *D. viscosa* is described in a large number of scientific papers and the main components of the essential oil obtained from different countries and regions are listed in the work of Zouaghi et al. [8]. Moreover, the phytochemical diversity of this plant has been described in detail by Grauso et al. [11], where all the compounds identified by different authors have been listed in view of explaining the antimicrobial, nematicidal and insecticidal activity of this versatile plant. Although *D. viscosa* is widely distributed along the Adriatic coast, to our knowledge, there are no data on the composition of volatile compounds in the essential oil and hydrosol of Croatian *D. viscosa*. Therefore, the first objective of this work is to determine the phytochemical composition, especially since we have not found any data on the hydrosol composition of *D. viscosa* from other regions either. Compared to essential oils, hydrosols contain more polar volatile compounds that are soluble in water [12], and we assume that these aqueous solutions are underestimated as mixtures of biologically active compounds that can be used as harmless natural products. Moreover, plant-derived natural products are environmentally friendly and often have a new mechanism of action that can overcome developed resistance [6]. Among the prominent biological effects described for this wild species [7,13,14,15], an interesting finding is the use of compounds from *D. viscosa* as a natural additive of polylactic acid, a biodegradable thermoplastic polymer, with the aim of modulating its physicochemical properties and achieving a bio-based packaging system [6]. Therefore, the present results on the analysis of the chemical composition of the essential oil and hydrosol of *D. viscosa* may be of great value for future studies and potential applications.

Recently, with the outbreak of a pandemic that we are still facing, the use of disinfectants in daily life has greatly increased, highlighting the need for natural disinfectants and antimicrobial products. An overview of the scientific literature revealed diverse reports of antimicrobial effects of *D. viscosa*, based on the variation in the chemical composition of the distillates, oils, and extracts tested, as well as the antimicrobial susceptibility assays chosen [11]. Ethanolic extract of *D. viscosa* leaves and flower buds showed antimicrobial activity against ATCC and foodborne isolates, with *Candida albicans* ATCC 10231 being the most sensitive strain [16]. We investigated the antimicrobial potential of the essential oil and, for the first time, hydrosol of *D. viscosa* by targeting thirteen opportunistic pathogens associated with common skin and wound infections and emerging food spoilage microorganisms.

Natural products with antiproliferative effects on tumor cells are the focus of modern medicine, with the goal of reducing the harmful effects of synthetic agents on healthy cells. Many studies have shown that *D. viscosa* is a plant with anti-cancer potential. Ozkan et al. [17] tested *D. viscosa* extracts on MCF-7 and T98-G cancer cells. The methanol extract showed a significantly stronger antiproliferative effect on both cell lines compared to the aqueous extract. Messaoudi et al. [18] also investigated the cytotoxic effect of ethanol and ethyl acetate extract of *D. viscosa* on two breast cancer cell lines. Both extracts inhibited the division of the tested cell lines after 72 h of treatment. The ethyl acetate extract showed higher cytotoxic activity, which the authors attributed to the synergistic effect of three dominant compounds: tomentosine, inuviscolide, and isocostic acid. Benbacer et al. [19] demonstrated a cytotoxic effect of *D. viscosa* extracts on two cervical cancer cell lines, in a manner that promoted apoptosis. Similar to the previous study, the key compound responsible for the cytotoxic activity is tomentosine, a sesquiterpene lactone that has been shown to be an extremely good anticancer agent [19,20]. We investigated the antiproliferative potential of *D. viscosa* volatiles on tree cancer cell lines: HeLa, HCT116, and U2OS. The possible mechanism of the antiproliferative activity of hydrosol was evaluated in relation to changes in intracellular GSH level since glutathione plays one of the most important roles in endogenous cell defense against oxidative stress.

Essential oils and other plant extracts have been used against a range of plant diseases caused by phytopathogenic bacteria, fungi, plant-parasitic nematodes, and parasitic and non-parasitic weeds [6]. Previous studies describe the activity of plant volatiles as natural antiphytoviral compounds [21,22,23,24,25,26,27,28,29]. The antiphytoviral activity of *D. viscosa* extracts has not been tested so far. Based on the chemical composition of essential oil and hydrosol, we assumed that this weed plant could be a readily available natural antiphytoviral agent. Indeed, the antiphytoviral activity of essential oils and hydrosols is of particular interest today from a green chemistry perspective. Low or no toxicity to non-target organisms and the possibility of obtaining them from renewable sources are just some of the advantages associated with the use of natural-based products in crop protection.

## 2. Results and Discussion

### 2.1. Gas Chromatography and Mass Spectrometry (GC-MS) Analysis of the Free Volatile Compounds from Essential Oil and Hydrosol

In this work, free volatile compounds were isolated from dried aerial parts of *D. viscosa* by water distillation in a Clevenger-type apparatus. The lipophilic (essential oil) and hydrofracture (hydrosol) collected from the inner tube of the Clevenger apparatus differ in chemical composition and biological activity due to the difference in solubility of the volatile compounds. In addition to water distillation, some authors have performed steam distillation, solvent extraction, and extraction by ultrasonic distillation from plant material of the genus Inula [8,9,10]. They obtained significant differences in the yield and composition of the essential oil [8]. In our study, the volatile compounds in the pentane layer (essential oil) and volatile compounds in the aqueous phase (hydrosol) were analyzed. The total oil yield was 0.09%, based on the dry weight of the samples. The composition and relative amounts of the compounds in both layers are shown in Table 1. Lipophilic compounds dissolved in pentane were analyzed by GC-MS. GC-MS analysis of the aqueous layer identified the more hydrophilic volatile components. Components that are soluble in both water and organic solvents were detected in the water and pentane layers (Table 1). Thus, GC-MS analysis of both phases, coupled with HPLC analysis of the hydrosol, provides us with a more complete phytochemical composition of the volatiles of this plant species. Haoui et al. [10] found that monoterpenes were the major chemical class of the essential oil of *D. viscosa* from Turkey and Algeria, while the class of oxygenated sesquiterpenes predominated in the plants from Spain, Italy, France, and Jordan [10]. In our study, twenty compounds, divided into six classes, and seventeen compounds, divided into three classes, were identified in the essential oil (EO) and hydrosol, accounting for 96.74% and 96.90% of the total oil and hydrosol composition, respectively. In terms of compound classes, the oxygenated monoterpenes dominate in the EO and hydrosol samples, accounting for 53.41% and 81.85% of the total composition, respectively. In addition to the oxygenated monoterpenes *1,8*-cineole identified as the dominant compound in the oil (16.41%) and *α*-terpinyl acetate (13.92%), the oxygenated sesquiterpenes caryophyllene oxide (15.14%) and *α*-muurolol (13.75%) stand out in the overall composition of the oil of *D. viscosa* (Table 1). *1,8*-Cineole (18.55%) represents the second most abundant component in the total hydrosol composition, and the compounds *α*-muurolol and caryophyllene oxide were also identified as frequent compounds in the hydrosol composition with proportions of 10.25% and 3.24%, respectively (Table 1). The oxygenated monoterpene *p*-menth-1-en-9-ol dominated the overall hydrosol composition (29.93%), while this compound was not detected in the oil composition. Linalool (11.67%) and *cis*-sabinene hydrate (10.97%) were also identified at a high percentage in the hydrosol and are also among the abundant components in the essential oil composition with proportions of 6.62% and 4.23%, respectively (Table 1). The oil contains a total of 30.11% oxygenated sesquiterpenes, with caryophyllene oxide (15.14%) and *α*-muurolol (13.75%) being the dominant compounds in this class and cyperotundone (1.22%) being less abundant. Madani et al. [30] compiled a table of the main components of the essential oils of *D. viscosa* from Algeria, Jordan, Italy, Turkey, Spain, and France. The composition of the oil of *D. viscosa* from Sardinia is most similar to the composition of the oil of Croatian *D. viscosa* in terms of caryophyllene oxide content. Caryophyllene oxide is also the main component obtained by water distillation from the leaves of this species from Algeria and Tunisia [30,31]. The fatty acid and hydrocarbon groups represent less than 6% of the total oil (Table 1). Differences in the composition of free volatiles of the species *D. viscosa* are influenced by population diversity, the time of collection of the plant material, and isolation techniques. We identified volatiles from two phases and, as shown in Table 1, some compounds were detected in both the lipophilic and aqueous phases, but some were identified in only one phase. This approach has given us a more complete insight into the chemical composition and potential application of specialized metabolites of the species *D. viscosa*.

### 2.2. HPLC Analysis of Hydrosol

Although hydrosols are important by-products of essential oil distillation, their chemical composition is generally analyzed relatively rarely. To detect more polar components of *D. viscosa* dissolved in the hydrosol, we subjected it to high-performance liquid chromatography (HPLC) in addition to GC-MS analysis. HPLC analysis of the hydrosol of *Veronica saturejoides* detected vanillin, cinnamic acid, and protocatechuic acid (synonim *3,4*-dihydroxybenzoic acid), the latter being the most abundant compound with an average concentration of 7.33 mg/L [33]. Beara et al. [34] also found significant amounts of protocatechuic acid in 70% aqueous acetone extracts of *Veronica teucrium* and *V. jacquinii*. Stojković et al. [35] described protocatechuic acid as the main compound in water extracts of *V. montana***,** while rosmarinic and caffeic acids were detected in hydrosol of *Poliomintha longiflora*, Mexican oregano [36]. Based on the retention times of standards and spiking samples, four phenolic acids and the flavonoid luteolin were identified in hydrosol of *D. viscosa* (Table 2). Together, these compounds account for more than 80% of the chromatogram area. The dominant phenolic acid with a concentration of 62.24 mg/L is *3,4*-dihydroxybenzoic acid (protocatechuic acid). This benzoic acid derivative is characterized by its antioxidant, anti-inflammatory [37], and antitumor activities [38]. Other phenolic compounds, cinnamic acid and its derivatives caffeic acid and *o*-coumaric acid are represented with much lower concentrations in the hydrosol composition (Table 2).

### 2.3. Wide-Spectrum Antimicrobial Activity

The antimicrobial activity of the hydrosol and essential oil was evaluated using a microdilution assay for a variety of human opportunistic pathogens associated with skin and wound infections, as well as foodborne pathogens. Bacterial and fungal growth was not affected by a 25% dilution of *D. viscosa* hydrosol. The essential oil was found to be effective against all the microorganisms tested as shown in Table 3. The essential oil inhibited bacterial growth at minimal inhibitory concentrations (MICs) ranging from as low as 0.09 mg/mL to 5.62 mg/mL, and fungi at MIC_50_ of 0.09 mg/mL and 2.81 mg/mL. Although the MIC_50_ values recorded for fungi were species-specific, the MIC_90_ of 5.62 mg/mL of essential oil was consistent for both yeast and mold strains. Of note, *C. albicans* is a commensal on human skin and mucoses but can cause infections ranging from superficial infections of the skin to life-threatening systemic infections [39]. Food-spoilage and food-poisoning mold *Aspergillus niger* is ubiquitous in the environment but has been implicated in serious opportunistic infections of humans, particularly pulmonary and cutaneous aspergillosis [40].

The essential oil strongly inhibited the growth of all tested bacteria, regardless of their Gram discrimination, and showed a strong and concentration-dependent bactericidal effect mostly at dosages of MICs. The most profound effect was recorded against *S. pyogenes* ATCC 19615, the clinical isolate of *Streptococcus agalactiae*, and the foodborne isolate of *Clostridium perfringens*, killing them at a dose of only 0.09 mg/mL of essential oil. All three bacterial species contribute significantly to skin and soft tissue infections in adults [41]. Moreover, *S. aureus* ATCC 29213 and methicillin-resistant *S. aureus* (MRSA) clinical strain was killed by dilutions of 2.81 and 5.62 mg/mL of essential oil, respectively (Table 3). Of note, *S. aureus* is one of the most common pathogens of nosocomial and community-associated infections worldwide [41]. This bacterium commonly causes skin, soft tissue, and bloodstream infections, 70% of which are due to MRSA strains that are resistant to almost all commercially available β-lactam and other classes of antibiotics [42]. Moreover, the MIC and MBC value of essential oil against *Acinetobacter baumannii* was 5.62 mg/mL. Notably, this pathogen deserves particular attention as one of the most common agents of various nosocomial infections, such as ventilator-associated pneumonia, urinary tract infections, bacteremia, and complicated skin and soft tissue, abdominal and central nervous system infections [43]. These infections are particularly difficult to treat due to their intrinsic and acquired resistance mechanisms, which places them in the group of multidrug-resistant ESKAPE pathogens (*Enterococcus faecium*, *S. aureus*, *Klebsiella pneumoniae*, *A. baumannii*, *Pseudomonas aeruginosa*, and *Enterobacter* spp.) with high medical priority [44].

Ali-Shtayeh et al. [45] reported that the water extract of *D. viscosa* was active against *C. albicans* by disc-diffusion method, while Al-Masri et al. [46] demonstrated antifungal activity against *Botryis cinerea* in terms of reduction of mycelium growth and germination when the hydrodistillation of *D. viscosa* was applied in combination with a low dose of the fungicide iprodione. Moreover, a number of studies reported stronger activity of alcohol extracts of *D. viscosa*, mainly methanol and ethanol, on both fungal and bacterial strains [11]. On the other hand, the antimicrobial activity of the essential oil of *D. viscosa* has been sparsely studied. Only Blanc et al. [47] demonstrated an antifungal effect of the essential oil (obtained by hydrodistillation as in our study) against the pathogenic and food-poisoning fungi *Aspergillus fumigatus*, *A. niger*, *C. albicans*, *Cladosporidium cladosporioides*, and *Cryptococcus neoformans*. Other authors used steam distillation to obtain the essential oil, which showed different antimicrobial activities ranging from no observed antifungal activity [48] to inhibition up to 84.11% [49].

Overall, the antimicrobial activity of the essential oil found in our study, in contrast to the hydrosol tested, is probably related to the higher concentration of oxygenated sesquiterpenes found in the oil (Table 1), compared to the hydrosol. In this context, the content of caryophyllene oxide, which was very abundant in the oil compared to the hydrosol, is of particular importance. This component has already been described as a potent antimicrobial substance when present in higher concentrations in *D. viscosa* hydrodistillation [8] and essential oil [31]. It should also be noted that the synergy between essential oil compounds may also contribute to the broad-spectrum antimicrobial activity shown in our study, as previously suggested by other authors [8].

### 2.4. Antiproliferative Activity

**Figure 1:**
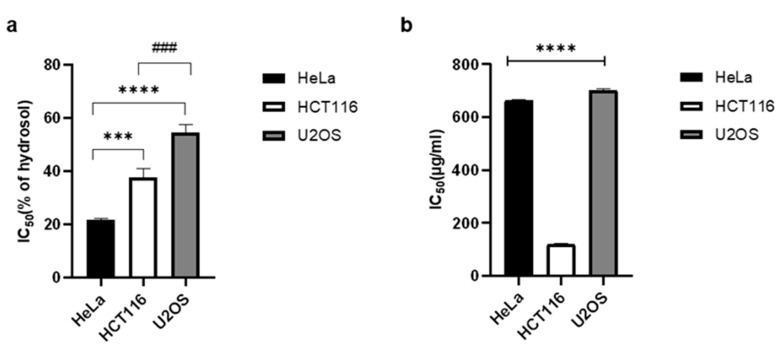
Antiproliferative activity of *Dittrichia viscosa* hydrosol (**a**) and essential oil (**b**) on HeLa, HCT116, and U2OS cancer cell lines assessed by MTS-based cell proliferation assay. Statistical significance was determined by one-way ANOVA, followed by Turkey’s multiple comparisons test. IC_50_ values are the means of three independent experiments. SD values are indicated with error bars. Statistically significant difference between HeLa and HCT116 cells is marked with *** *p* < 0.001, between HeLa and U2OS is marked with **** *p* < 0.0001 and between HCT116 and U2OS is marked with ^###^ *p* < 0.001.

Recently, much research has been done with the aim of finding natural chemotherapeutic agents. Numerous studies have shown that extracts from different parts of *D. visocosa* have promising cytotoxic activity [18,19,50,51,52,53,54,55]. In this study, we tested for the first time the antiproliferative activity of the volatile compounds in the essential oil and a hydrosol of *D. viscosa*. The essential oil showed potent antiproliferative activity on all three cancer cell lines used: HeLa, HCT116 and U2OS (IC_50_ 0.66 mg/mL, 0.12 mg/mL and 0.7 mg/mL, respectively). The hydrosol significantly inhibited the division of cancer cells with IC_50_ values, 21.70% for HeLa cells, 37.73% for HCT116, and 54.51% for U2OS (Figure 1).

Ozkan et al. [17] showed that the methanol extract of areal parts of *D. viscosa* had a stronger antiproliferative effect than the aqueous extract on two tested cell lines, MCF-7 (human breast adenocarcinoma) and T98-G (human brain tumor). In the study by Benbacer et al. [19], methanol extract of *Dittrichia* also showed significant inhibition of proliferation of human cervical cancer cells, HeLa and SiHa. The main mechanism of action involves the induction of programmed cell death. The ethanol extract of *D. viscosa* flowers inhibited the growth and proliferation of Vero cells, with an IC_50_ value of 202.43 µg/mL. The methanol fraction had excellent antiproliferative activity on MCF-7 cells with an IC_50_ value of 15.78 µg/mL [50,54].

Numerous compounds present in *D. viscosa* extracts have shown significant biological activities [56,57,58]. These mainly include flavonoids such as nepetin, hispidulin, and methylquercetin. In the present study, GC-MS analysis revealed the presence of monoterpenoids *p*-menth-1-en-9-ol and *1,8*-cineole as the dominant compounds in the hydrosol (Table 1). Oxygenated monoterpenes have been previously described as compounds with anticancer activity [59]. Moteki et al. [60] reported the cytotoxic activity of *1,8*-cineole in leukemia cancer cells. Although the mechanism of cytotoxic activity is not fully elucidated, the authors showed that the suppression of leukemia cell growth was associated with the induction of apoptosis. Murata et al. [61] also showed that the main mechanism of inhibition of colorectal cell proliferation was apoptosis. Treatment with *1,8*-cineole activated p38 and dephosphorylated Akt, leading to activation of caspase-3 and induction of apoptosis. A study conducted on three cancer cell lines (MCF7, A2780, and HT29) and one normal fibroblast cell line (MRC5) showed that *1,8*-cineole acts selectively and causes a remarkable dose-dependent inhibition of the growth of cancer cells but not of healthy fibroblasts [62]. Thus, *1,8*-cineole emerges as a promising, safe, and potent chemotherapeutic agent for the treatment of various cancers. In addition, *3,4*-dihydroxybenzoic acid or protocatechuic acid (PCA), which is present in a variety of fruits, vegetables, and a number of medicinal plants [63] as well as in the hydrosol of *D. viscosa* (Table 2), possesses a wide range of biological activities such as antioxidant activity, antiviral, anti-inflammatory, anticancer, and many others [64,65,66,67,68,69]. Due to its chemical structure, it acts as an excellent antioxidant. It also has the possibility of prooxidant activity, which probably plays an important role in inhibiting the proliferation of cancer cells. Thus, the activity of hydrosol on cancer cells could be related to the high content of this phenolic compound in addition to *1,8*-cineole. Previous studies have shown a significant antiproliferative effect of *3,4*-dihydroxybenzoic acid on immortalized breast cells HBL 100, breast cancer cells PC14 and promyelocytic leukemia cells HL-60 [65,66,67,68] and liver cancer cell line HepG2 [69], possibly by generating oxygen free radicals that act as signaling molecules and affect genes involved in cell cycle regulation and apoptosis [70].

The results of our study confirmed that in addition to the previously demonstrated antiproliferative activity of various extracts of *D. viscosa*, hydrosol tested for the first time exhibited very significant antiproliferative activity on cancer cell lines. The anti-cancer potential of *D. viscosa* volatiles should be investigated on other cell lines, focusing on extracts from different parts of the plant, with the aim of finding new active molecules that could be used in the treatment of different types of cancer.

### 2.5. Glutathione (GSH) Assay

The effect of *D. viscosa* hydrosol on intracellular GSH level was measured using Ellman’s reagent. A change in GSH level is important for assessing toxicological responses and is an indicator of oxidative stress, possibly leading to apoptosis and cell death. The significant amount of *3,4*-dihydroxybenzoic acid in hydrosol composition (Table 2) and its dual role of acting as both antioxidant and prooxidant, as well as its important role in the proliferation of cancer cells and prevention of carcinogenesis [71], prompted us to investigate the effect of hydrosol treatment on GSH level in HeLa cells. Glutathione is considered to be a very important factor in regulating carcinogenic mechanisms in cancer cells [72]. In contrast to its protective role in healthy cells, where it is crucial for neutralizing carcinogens, elevated GSH levels in tumor cells are associated with tumor progression and increased resistance to chemotherapeutic agents [73]. In recent years, several novel therapies targeting the antioxidant GSH system in tumor cells have been developed to achieve better response and reduced drug resistance. HeLa cells treated with the hydrosol for 1 h (IC_50_ from MTT measurements) showed a significant reduction in GSH level compared to untreated control cells (Table 4). The reduced GSH level indicates the direction of the cellular response in oxidative homeostasis, suggesting oxidative stress as a possible mechanism of the antiproliferative effect of hydrosol on tumor cells.

### 2.6. Antiphytoviral Activity

**Figure 2:**
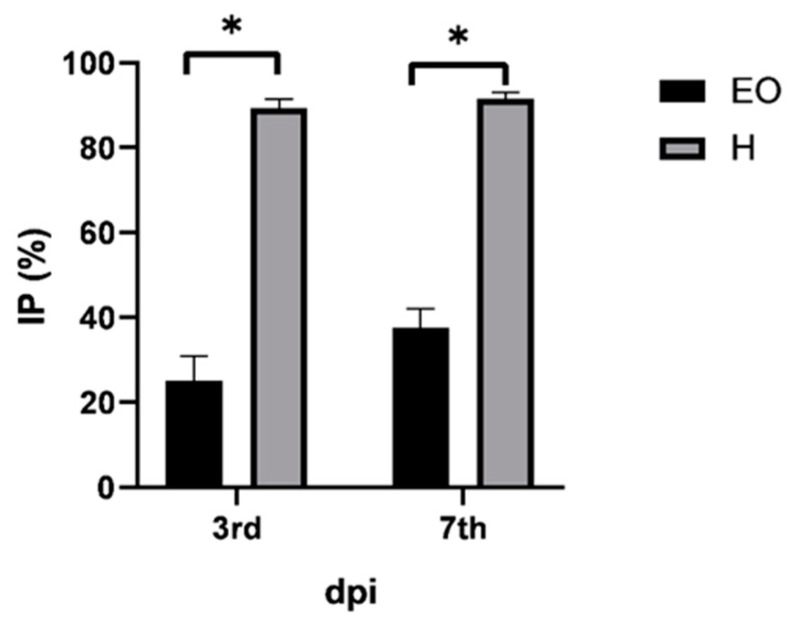
Percentage of inhibition (IP) of lesions on leaves of treated *Datura stramonium* plants inoculated with tobacco mosaic virus compared to control plants on days three and seven postinoculation (dpi). Prior to inoculation, *D. stramonium* plants were treated with essential oil (EO) or hydrosol (H) of *Dittrichia viscosa* for two consecutive days. Error bars show the standard deviation of triplicate analyses; significant differences were marked with *.

The search for substances of natural origin with antiphytoviral activity is particularly important today to support biological production and the replacement of synthetic chemicals with natural agents. Scientific literature reports that the application of water extracts of *D. viscosa* in combination with a low dose of the effective fungicide iprodione may be a viable way to reduce the severity of gray mold disease [46]. A mixture of acetone and *n*-hexane extract of *D. viscosa* emulsified in water effectively controlled downy mildew of cucumber, late blight of potato or tomato, powdery mildew of wheat, and rust of sunflower [74]. Based on the phytochemical composition of the essential oil and hydrosol (Table 1 and Table 2), we hypothesized that the biological activities of *D. viscosa* extracts could be extended in terms of antiphytoviral activity. The activity of both lipophilic and hydrophilic extracts of *D. viscosa* on the defense response of local host plants to tobacco mosaic virus (TMV) infection was investigated. TMV is a model virus in plant virology and a very important pathogen of agricultural crops causing significant yield losses. In addition to the antiphytoviral activity of essential oils of aromatic plants [21,22,23,24,25,26,27,28,29], the activity of hydrosol of *Hypericum perforatum* ssp. *veronense* was recently demonstrated [26], showing that hydrosols are a readily available natural source of bioactive compounds that can be used for plant protection against viral pathogens. Moreover, considering that hydrosols are a by-product of the essential oil distillation process, it is clear that the use of all products of this process is environmentally and biologically desirable. Although both essential oil and hydrosol-treated plants significantly reduced the number of local lesions compared to control plants (Table 5), the percentage inhibition of local lesions was more pronounced in hydrosol-treated plants (Figure 2). On the third day post-inoculation, the inhibition of lesions on the leaves of the essential oil- and hydrosol treated plants was 25.1% and 89.3%, respectively, and on the seventh day after inoculation, this inhibition was 37.5% and a promising 91.5%, respectively (Figure 2). Based on the results (Table 5, Figure 2) and the fact that our preliminary study showed that simultaneous inoculation of hydrosol and virus did not reduce the number of local symptoms, we concluded that pretreatment with hydrosol activates the plant defense response and increases resistance to viral pathogens. Considering that salicylic acid (*2*-hydroxybenzoic acid) is one of the most important endogenous signals in the activation of plant defense response [75], we suggest that the antiviral activity of the hydrosol of *D. viscosa* may be related to the high content of benzoic acid derivative, namely *3,4*-diydroxybenzoic acid (Table 2). In addition, it is also possible that other components contained in the hydrosol (Table 1 and Table 2) have synergic effects and activate plant signaling pathways leading to increased resistance to viral infections. The reported antiphytoviral activity of both the essential oil and hydrosol deserves more detailed analysis in the future and opens new areas of research regarding this unexplored bioactivity of *D. viscosa*. Further studies are required to evaluate the efficacy against viral diseases under field conditions.

## 3. Materials and Methods

### 3.1. Herbal Material

Plants were harvested from a ruderal habitat at the Žnjan locality, Split, Croatia (43°30′34.2″ N, 16°28′33.3″ E), at the full flowering stage from September 2018 to September 2020. The identity of the plant was confirmed by Prof. Mirko Ruščić based on the literature [3,4]. Voucher specimens of the plant material were deposited at the Faculty of Science, Department of Biology, University of Split, Split, Croatia. The samples were air-dried in a single layer in a well-ventilated room for two weeks and protected from direct sunlight. The dried plant material was packed in paper bags and stored in a dry place protected from light until hydrodistillation. The randomized mixture of these samples was used for hydrodistillation. Three isolations of essential oil and hydrosol were carried out.

### 3.2. Hydrodistillation and Analyses of Free Volatile Compounds

One liter of water was added to 100 g of the dried plant material in the flask of the Clevenger apparatus. Water (35 mL) and pentane (VWR Chemicals, Radnor, PA, USA) were added to the inner tube of the Clevenger apparatus. The hydrodistillation lasted for 3 h. Finally, the fractions of lipophilic (essential oil, EO) and hydrophilic volatile compounds (extracted into pentane and water fractions) were collected separately from the apparatus. The excess pentane was evaporated to calculate the oil yield. The oil was then resuspended, and the final essential oil concentration was 90 mg/mL. This stock solution was stored at −20 °C. The hydrosol was collected from the apparatus and stored at +4 °C. Both phases were analyzed by GC and GC-MS. Gas chromatography (GC) was performed using a gas chromatograph (model 3900; Varian Inc., Lake Forest, CA, USA) equipped with a flame ionization detector (FID) and a mass spectrometer (model 2100T, Varian Inc., Lake Forest, CA, USA). The chromatographic conditions for nonpolar (VF-5 ms, 30 m × 0.25 mm × 0.25 μm, Palo Alto, CA, USA) and polar (CP-Wax 52 CB, 30 m × 0.25 mm × 0.25 μm, Palo Alto, CA, USA) capillary columns were as described in the work of Vuko et al. [26]. The injected volume of essential oil was 2 μL. For the hydrophilic fraction, the injection was performed with a headspace injection needle, and there was no split ratio (splitless mode). The 2 g of hydrosol was added to the glass bottle and sealed with a metal cap with a septum. The headspace needle was injected into the glass bottle sealed with a metal cap with a septum. The glass bottle was first placed in 40 °C water with the hydrosol sample and allowed to stand without the needle for 20 min to allow the volatile compounds to evaporate from the water. The needle was then injected and left for 20 min to allow the volatile compounds to adsorb onto the resin needle. The injection needle was then inserted into a GC inlet and left there for 20 min to ensure that all volatile compounds were reabsorbed by the resin into the injection liner.

The individual peaks for all samples were identified by comparing their retention indices of *n*-alkanes with those of authentic samples and literature [32]. The results for all samples were measured in three independent analyzes and expressed as the percentage (%) of each compound (Table 1).

### 3.3. High-Performance Liquid Chromatography (HPLC)

Phenolic compounds were separated on an Ultra Aqueous C18 column (250 mm × 4.6 mm, 5 mm; Restek; Bellefonte, PA, USA) maintained at 25 °C. A gradient consisting of solvent A (water/phosphoric acid, 99.8:0.2, *v*/*v*) and solvent B (methanol/acetonitrile, 50:50, *v*/*v*) was applied at flow rate of 0.8 mL/min as follows: 0.5 min 96% A and 4% B; 40 min 50% A and 50% B; 45 min 40% A and 60% B; 60 min 0% A and 100% B; 70 min 96% A and 4% B; 80 min 96% A and 4% B. The injection volume was 20 µL and the signal was monitored at 280 nm. Phenolic acids were identified by comparing their retention times (Supplementary Data) with corresponding standards (Sigma-Aldrich, St. Louis, MO, USA) analyzed under the same conditions and by spiking samples. The compounds were quantified using external standard calibration curves. Concentrations of compounds are expressed as mg of compound per L hydrosol (mg/L).

### 3.4. Microbial Strains and Culture Conditions

To evaluate antimicrobial activity, hydrosol and essential oil of *D. viscosa* were tested against thirteen strains of human opportunistic pathogens and food spoilage microorganisms. Antimicrobial testing included Gram-negative *Escherichia coli* ATCC 25922 and *Acinetobacter baumannii* ATCC 19606, and eight Gram-positive species: *Staphylococcus aureus* (including ATCC 29213 and a methicillin-resistant *S. aureus* clinical strain MRSA-1), *Staphylococcus epidermidis* human isolate, *Streptococcus pyogenes* ATCC 19615, *Streptococcus agalactiae* clinical isolate, *Enterococcus faecalis* ATCC 29212, *Listeria monocytogenes* ATCC 19111 (1/2a), and food-borne isolates of *Bacillus cereus* and *Clostridium perfringens* [76,77]. The multidrug-resistant clinical MRSA strain was obtained from the University Hospital Centre Split, Croatia [78]. The antifungal activity was assessed against the opportunistic yeast *Candida albicans* ATCC 90029 and the environmental isolate *Aspergillus niger*. Antibiotic susceptibility testing was carried out using Etest (AB Biodisk, Solna, Sweden) and the VITEK 2 system (bioMérieux, Craponne, France). Microorganisms were stored at −80 °C and subcultured on tryptic soy agar (TSA; Biolife, Milan, Italy) or Sabouraud dextrose agar (SDA; Biolife, Milan, Italy) before testing.

### 3.5. Broth Microdilution Assays

The antimicrobial activity was tested using broth microdilution assay according to the guidelines of The European Committee on Antimicrobial Susceptibility Testing (EUCAST) for bacteria and fungi [79]. Sabouraud dextrose broth (SDB; Biolife) was used for fungal growth.

Two-fold dilutions of essential oil (ranging from 22.5 to 0.02 mg/mL) and hydrosol (ranging from 25% to 0.024%) were tested. Experiments were carried out in 96-well microtiter plates as previously described [76,77]. Briefly, bacterial cultures were exponentially grown in Mueller-Hinton broth (MHB; Biolife), adjusted spectrophotometrically to reach 10^6^ CFU/mL, added to serial two-fold dilutions of essential oil and hydrosol in a final volume of 100 µL per well, and further incubated at 37 °C for 18 h. In the case of fungi, an inoculum of approximately 2.5 × 10^5^ CFU/mL of spores/conidia were added to the wells and incubated at 35 °C for 24 h (*C. albicans*) and 48 h (*A. niger*). The minimal inhibitory concentration (MIC) was determined as the lowest concentration showing no visible bacterial growth (turbidity) in the wells. For minimal bactericidal concentration (MBC) determination, aliquots were taken from the wells corresponding to the MIC, 2 × MIC, and 4 × MIC, and plated on MHA plates. After incubation at 37 °C for 18 h, the MBC value was recorded as the lowest concentration causing ~99.9% killing of the starting inoculum.

In the case of fungi, the aliquots taken from the wells were plated on SDA and incubated for 24 and 48 h at 35 °C. After colony counting, MIC_50_ and MIC_90_ endpoints were recorded as the lowest concentrations that inhibited 50% and 90% of fungal growth compared to the control. All tests were carried out in triplicate.

Data on the susceptibility of the microbial strains used in this study have been published previously [76,80].

### 3.6. Antiproliferative Analysis

The antiproliferative activity of essential oil and hydrosol of *D. viscosa* was determined on cancer cells of cervical cancer cell line (HeLa), human colon cancer cell line (HCT116), and human osteosarcoma cell line (U2OS) using the MTS-based CellTiter 96^®^ Aqueous Assay (Promega) according to the procedure described in our previous papers [26,77]. Cells were kindly provided to us by prof. Janoš Terzić from the School of Medicine, University of Split. Cells were grown in a CO_2_ incubator at 37 °C and 5% CO_2_ until they reached 80% confluency. They were counted using the automatic handheld cell counter (Merck, Darmstadt, Germany), seeded in 96-well plates, and treated with serially diluted essential oil and hydrosol. Cells were further grown for 48 h, after which 20 µL of MTS tetrazolium reagent (Promega, Madison, WI, USA) was added to each well. After 3 h of incubation at 37 °C and 5% CO_2_, absorbance was measured at 490 nm using a 96-well plate reader (Bio-Tek, EL808, Winooski, VT, USA). Measurements were performed in four replicates for each concentration and IC_50_ values were calculated from three independent experiments using GraFit 6 data analysis software (Erithacus, East Grinstead, UK).

### 3.7. Glutathione (GSH) Assay

Intracellular GSH changes were measured using Ellman’s reagent (DTNB; Sigma-Aldrich, St. Louis, MO, USA) employing the protocol proposed by Tan et al. [81]. The absorbance was measured at 405 nm using a microplate reader (BioSan, Riga, Latvia). The concentration of free thiols in the samples was calculated using a GSH (Sigma-Aldrich, St. Louis, MO, USA) standard curve.

### 3.8. Antiphytoviral Activity

#### 3.8.1. Virus and Plant Hosts

Leaves of *Nicotiana tabacum* L. cv. Samsun systemically infected with tobacco mosaic virus were used to prepare the virus inoculum as described by Vuko et al. [26]. Leaves of the local host *Datura stramonium* L. were dusted with silicon carbide (Sigma-Aldrich, St. Louis, MO, USA) prior to virus inoculation, and the inoculum was diluted with inoculation buffer to obtain 5–30 lesions per inoculated leaf. The experiments were carried out when the plants grew to the 5–6 leaf stage. Care was taken to ensure that the experimental plants were as uniform in size as possible.

#### 3.8.2. Antiphytoviral Activity Assay

Essential oil (0.045 mg/mL) or hydrosol (undiluted) were applied as a spray solution to the leaves of local host plants on two consecutive days prior to virus inoculation. The plants were then rubbed with virus inoculum and the antiviral activity of the essential oil and hydrosol was evaluated by the percentage inhibition towards the number of local lesions on the leaves of the treated and control plants as described by Vuko et al. [26].

### 3.9. Statistical Analysis

Statistical analysis was performed in GraphPad Prism Version 9. All data are expressed as mean ± SD (*n* ≥ 3). Statistical significance was assessed by *t*-test (free volatile compounds, inhibition of local lesions, and GSH assay), one-way ANOVA (number of local lesions), and one-way ANOVA followed by Turkey’s multiple comparison test (antiproliferative activity). Differences were considered significant at * *p* < 0.05.

## 4. Conclusions

*1,8*-Cineole and caryophyllene oxide as well as *p*-menth-1-en-9-ol, *1,8*-cineole, and *3,4*-dihydroxybenzoic acid are the most abundant compounds in the lipophilic fraction (essential oil) and in the water fraction (hydrosol) of Croatian *Dittrichia viscosa* (L.) Greuter. The antimicrobial activity of the essential oil and the antiproliferative activity of both extracts shown in this study open new areas of research regarding the use of this plant species as a natural disinfectant and antiproliferative agent, which should be further confirmed by additional toxicological tests. The essential oil and hydrosol also showed antiphytoviral activity, suggesting that the volatiles of *D. viscosa* have the potential for the development of natural antiphytoviral preparations. The results presented in this study indicate the importance of further research on *D. viscosa*.

## Figures and Tables

**Table 1:** Phytochemical composition (% ± SD) of the essential oil (EO) and hydrosol (H) of *Dittrichia viscosa*.

Component	RI*	RI**	EO (Yield in %)	H (Yield in %)
*Monoterpene hydrocarbons*			0.71	1.56
*α*-Pinene *	938	1036	0.71 ± 0.01 ^a^	0.23 ± 0.01 ^b^
Sabinene	971	1115	-	0.28 ± 0.01
Myrcene	992	1145	-	0.62 ± 0.03
Limonene	1032	1204	-	0.43 ± 0.03
*Oxygenated monoterpenes*			53.41	81.85
*1,8*-Cineole	1030	1211	16.41 ± 0.01 ^b^	18.55 ± 0.01 ^a^
*cis*-Sabinene hydrate	1065	1561	4.23 ± 0.01 ^b^	10.97 ± 0.01 ^a^
Linalool	1099	1548	6.62 ± 0.01 ^b^	11.67 ± 0.01 ^a^
Borneol *	1176	1699	0.32 ± 0.01 ^a^	0.12 ± 0.05 ^b^
α-Terpineol	1186	1646	2.65 ± 0.01 ^a^	2.62 ± 0.01 ^b^
*β*-Cyclocitral	1223	1629	4.81 ± 0.01 ^a^	2.23 ± 0.01 ^b^
Bornyl acetate	1287	1591	2.71 ± 0.01 ^a^	0.67 ± 0.01 ^b^
*p-*Menth-1-en-9-ol	1294	1915	-	29.93 ± 0.01
α-Terpinyl acetate	1349	1685	13.92 ± 0.01 ^a^	1.31 ± 0.01 ^b^
Cyclohexene, 1,5,5-trimethyl-6-methylene	1364	-	-	2.02 ± 0.01
(*E*)-Isoeugenol	1446	2314	1.74 ± 0.01	1.76 ± 0.01
*Sesquiterpene hydrocarbons*			7.26	-
a*llo*-Aromadendrene	1465	1662	2.34 ± 0.01	-
*β*-Bisabolene	1494	1729	4.31 ± 0.01	-
*δ*-Cadinene	1517	1754	0.61 ± 0.07	-
*Oxygenated sesquiterpenes*			30.11	13.49
Caryophyllene-oxide *	1581	1955	15.14 ± 0.01 ^a^	3.24 ± 0.01 ^b^
α-Muurolol	1645	2181	13.75 ± 0.01 ^a^	10.25 ± 0.01 ^b^
Cyperotundone	1696	-	1.22 ± 0.01	-
*Fatty acids*			2.58	-
Hexadecanoic acid	1959	2913	2.58 ± 0.01	-
*Hydrocarbons*			2.67	-
Heneicosane *	2100	2100	0.42 ± 0.03	-
Docosane *	2200	2200	1.73 ± 0.01	-
Tricosane *	2300	2300	0.52 ± 0.01	-
*Total identification (%)*			96.74	96.90

Retention indices (RI) were determined relative to a series of n-alkanes (C_8_–C_40_) on capillary columns VF5-ms (RI*) and CP Wax 52 (RI**); RI, identification by comparison of RIs with samples listed in a homemade library, reported in the literature [32] and/or authentic samples; comparison of mass spectra with those in the NIST02 Wiley 9 mass spectral libraries; * co-injection with reference compounds; -not identified; SD standard deviation of triplicate analyzes; significant differences were determined using multiple *t*-tests^. a,b^ Mean values with different superscripts indicate a statistically significant difference between the data from EO and the H sample (*p* < 0.05).

**Table 2:** HPLC analysis of phenolic compounds.

Phenolic Compound	mg/L ± SD
*3,4*-dihydroxybenzoic acid	62.24 ± 2.72
caffeic acid	0.90 ± 0.06
*trans-o*-coumaric acid	0.39 ± 0.02
cinnamic acid	1.16 ± 0.01
luteolin	1.75 ± 0.13

**Table 3:** Antimicrobial activity of *Dittrichia viscosa* essential oil by microdilution assay.

Species	Strain Origin	Essential Oil (mg/mL) ^a^	
		MIC	MBC
Gram-positive bacteria			
*Staphylococcus aureus*	ATCC 29213	2.8	2.8
*Staphylococcus aureus*	Clinical/MRSA	5.6	5.6
*Staphylococcus epidermidis*	Human	1.4	1.4
*Streptococcus pyogenes*	ATCC 19615	0.09	0.09
*Streptococcus agalactiae*	Clinical	0.09	0.09
*Enterococcus faecalis*	ATCC 29212	1.4	2.8
*Listeria monocytogenes*	ATCC 19111 (1/2a)	2.8	2.8
*Bacillus cereus*	Food	0.7	0.7
*Clostridium perfringens*	Food	0.09	0.09
Gram-negative bacteria			
*Escherichia coli*	ATCC 25922	2.8	2.8
*Acinetobacter baumannii*	ATCC 19606	5.6	5.6
Yeast		MIC_50_	MIC_90_
*Candida albicans*	ATCC 90029	2.8	5.6
Molds		MIC_50_	MIC_90_
*Aspergillus niger*	Food	0.09	5.6

^a^ Two-fold dilutions of essential oil were tested in a range from 22.5 to 0.02 mg/mL by the microdilution method.

**Table 4:** GSH level (mmol/L) ±SD in hydrosol (H) treated HeLa cells compared to untreated cells.

	Control	H Treated
GSH level	0.287 ± 0.007 ^a^	0.187 ± 0.025 ^b^

^a, b^ Mean values with different superscripts indicate a statistically significant difference between the data (*p* < 0.05).

**Table 5:** Local lesion number (LLN) on leaves of treated *Datura stramonium* plants inoculated with tobacco mosaic virus and on leaves of control plants (C) on days three and seven post-inoculation (dpi). Prior to inoculation, *D. stramonium* plants were treated with essential oil (EO) or hydrosol (H) of *Dittrichia viscosa* for two consecutive days.

dpi		LLN ± SD
3rd	C	6.2 ± 0.2 ^a^
	EO	4.6 ± 0.2 ^b^
	H	0.7 ± 0.2 ^c^
7th	C	14.2 ± 2.4 ^a^
	EO	8.9 ± 2.0 ^b^
	H	1.2 ± 0.4 ^c^

SD, the standard deviation of triplicate analysis; significant differences were determined by one-way ANOVA. ^a, b, c^ Mean values with different superscripts indicate statistically significant differences between control and essential oil/hydrosol treatment data (*p* ˂ 0.05).

## Data Availability

All data is contained within the article.

## Supplementary Materials

The following are available online at https://www.mdpi.com/article/10.3390/plants10091837/s1, Table S1: Mass concentration range of the standards, the corresponding correlation coefficients (r^2^) and the retention time.
