# Supplementary material for: Not Only a Weed Plant—Biological Activities of Essential Oil and Hydrosol of Dittrichia viscosa (L.) Greuter
Source: Plants (Basel). 2021 Sep 4;10(9):1837. doi: 10.3390/plants10091837 (PMC8470837; doi:10.3390/plants10091837)
Supplement: Supplementary file 1 [file plants-10-01837-s001.zip › plants-1331794-supplementary.pdf]

Table S1. Mass concentration range of the standards, the corresponding correlation coefficients (r^2) and the retention time

**Phenolic compound**	**Working range (µg mL^{-1})**	**r^2**	**Retention time (min)**
*3,4-*dihydroxybenzoic acid	0.46-268.80	0.9999	20.76
caffeic acid	0.20-124.60	0.9999	28.04
trans-*o*-coumaric acid	0.13-80.20	0.9999	37.94
cinnamic acid	0.13-81.80	0.9996	46.00
luteolin	5.12-200.00	0.9997	49.09
